# Correction: computational design, synthesis, and assessment of 3-(4-(4-(1,3,4-oxadiazol-2-yl)-1*H*-imidazol-2-yl)phenyl)-1,2,4-oxadiazole derivatives as effective epidermal growth factor receptor inhibitors: a prospective strategy for anticancer therapy

**DOI:** 10.1039/d4md90040e

**Published:** 2024-10-15

**Authors:** Nilesh Raghunath Khedkar, Milind Sindkhedkar, Alex Joseph

**Affiliations:** a Novel Drug Discovery & Development, Lupin Research Park, Lupin Ltd. Pune-412115 India milindsindkhedkar@lupin.com; b Department of Pharmaceutical Chemistry, Manipal College of Pharmaceutical Sciences, Manipal Academy of Higher Education Manipal Karnataka-576104 India

## Abstract

Correction for ‘Computational design, synthesis, and assessment of 3-(4-(4-(1,3,4-oxadiazol-2-yl)-1*H*-imidazol-2-yl)phenyl)-1,2,4-oxadiazole derivatives as effective epidermal growth factor receptor inhibitors: a prospective strategy for anticancer therapy’ by Nilesh Raghunath Khedkar *et al.*, *RSC Med. Chem.*, 2024, **15**, 1626–1639, https://doi.org/10.1039/D4MD00055B.

The authors regret that the affiliation of one of the authors (Nilesh Raghunath Khedkar) was shown incorrectly in the original manuscript. The corrected affiliation is shown here.

The Royal Society of Chemistry apologises for these errors and any consequent inconvenience to authors and readers.

